# Changes in cerebral oxygenation during early postnatal adaptation in newborns delivered by vacuum extraction measured by near-infrared spectroscopy

**DOI:** 10.1186/1471-2431-14-21

**Published:** 2014-01-27

**Authors:** Tanja Karen, Martin Wolf, Rahel Nef, Daniel Haensse, Hans Ulrich Bucher, Gabriele Schulz, Jean-Claude Fauchère

**Affiliations:** 1 Division of Neonatology, Department Obstetrics & Gynecology, University Hospital Zurich, Frauenklinikstrasse 10, Zurich CH-8091, Switzerland

**Keywords:** Cerebral oxygenation, Near-infrared spectroscopy, Newborn, Vacuum delivery

## Abstract

**Background:**

Newborns delivered by vacuum extraction quite often show clinical signs of a hemodynamic compromise, which is difficult to assess in terms of severity. The conventional means to measure the hemodynamic status are not sensitive enough to appreciate the severity of general, and more specifically of cerebral circulatory imbalance. The aim was to study cerebral tissue oxygenation during postnatal adaptation in these infants using near-infrared spectroscopy.

**Methods:**

The tissue hemoglobin index (THI), tissue oxygenation index (TOI), arterial oxygen saturation (pre-ductal SaO_2_) and heart rate (HR) were recorded immediately after birth, and again after 12–24 hours of life in 15 newborns delivered by vacuum extraction due to fetal distress. A comparison with 19 healthy newborns delivered by elective cesarean section was performed.

**Results:**

Newborns delivered by vacuum extraction had significantly higher THI 10 to 15 minutes after birth. TOI and HR were significantly higher in the first 5 min and SaO_2_ in the first 10 minutes but then did not differ from those after cesarean section.

**Conclusion:**

Infants delivered by vacuum extraction following fetal distress show transient deviations in cerebral oxygenation and perfusion after birth which were not detectable after 24 hours.

## Background

The transition from fetal to extra-uterine life is characterized by a number of unique physiological changes within the first minutes to hours, or even days after birth. There is a substantial body of literature concerning the changes in arterial oxygen and carbon dioxide content, in pulmonary and systemic blood flow, the closure of the intra- and extra-cardiac shunts, and the changes in oxygen saturation immediately after birth [1-5]. These changes, together with the compression of the skull during delivery followed by decompression at the moment of birth, may be assumed to play important parts in the cerebral circulatory adaptation to neonatal life [1]. The mechanisms, which regulate the postnatal adaptation of cerebral blood flow are poorly understood [6-8]. Data about these changes, especially immediately after birth, are scarce [9-17]. Furthermore, data are lacking in infants, in whom the postnatal adaptation of the circulatory and respiratory systems do not occur smoothly. Because of the possibility of permanent central nervous damage, the assessment of cerebral circulation and oxygenation dynamics is essential during this critical phase. Abnormal labor, instrumental vaginal delivery or emergency cesarean section for fetal distress, have been associated with an increased risk of low Apgar scores, fetal acidosis, and moderate to severe neonatal encephalopathy [18-21]. The conventional means to assess the hemodynamic status in these infants such as heart rate, capillary refilling, pulse quality and non-invasive blood-pressure are not sensitive enough to accurately assess the magnitude of a hemodynamic imbalance. For instance, arterial blood pressure can remain normal in a situation of impaired cardiac output due to compensatory vasoconstriction and considering a mean arterial blood pressure equal to gestational age as a normal blood pressure within the first 24 – 48 hours of life.

Our true interest, however, is to obtain more precise information concerning cerebral tissue oxygenation, and even more important to assess the severity of cerebral hemodynamic compromise, and thereby to help the clinician to decide if a baby requires a therapeutic intervention.

Near-infrared spectroscopy (NIRS) is a validated tool to assess changes in oxygenated (O_2_Hb) and deoxygenated hemoglobin (HHb) in the brain, and thereby it directly measures cerebral tissue oxygenation. This tool has been extensively evaluated in preterm and term neonates undergoing intensive care, and it has been shown that NIRS is able to monitor cerebral hemodynamics in critically ill term and preterm infants [22,23].

The aim of this study was to determine the effect of vacuum extraction assisted vaginal delivery on the changes in cerebral perfusion and oxygenation in the first fifteen minutes of life and at 24 hours of life measured by near- infrared spectroscopy.

## Methods

### Setting and patients

15 newborn infants born at the University Hospital Zurich who were delivered by vacuum extraction due to fetal distress (abnormal cardiotocogram, meconium stained amniotic fluid) were eligible for this study. The exclusion criteria were a genetically defined syndrome, a congenital malformation, and absence of parental consent or poor quality NIRS signals. At our perinatal center, neonates born by vacuum extraction are routinely under the supervision of a neonatologist for the first 10 to 15 minutes after birth. The neonatal resuscitation was performed according to a standard protocol [24].

Immediately after birth the infants were placed on a resuscitation table under a radiant warmer and taken care of by a neonatologist. The head and the right arm were cleansed. Using a pulse oximeter (Covidien-Nellcor N-395, Boulder, Colorado, USA) attached to the right hand or wrist, both the arterial oxygen saturation (tcSaO_2_) and heart rate (HR) were recorded. When measured on the right hand or wrist, SaO_2_ is representative of the oxygen saturation reaching the brain. At the same time a near-infrared sensor (NIRO-300^TM^ Hamamatsu Photonics, Hamamatsu, Japan) was placed over the right forehead of the infant and the measurements of oxygenated (O_2_Hb) and deoxygenated hemoglobin (HHb), tissue oxygenation index ( TOI) and tissued hemoglobin index (THI) were performed until 15 minutes after birth. THI, which is calculated as the sum of O_2_Hb and HHb corresponds to cerebral blood volume (CBV) provided that the hematocrit remains constant [25]. The optodes were kept in place by an elastic bandage, and covered by a light-occluding cloth to prevent contamination of the near-infrared signal by external light. The NIRS sensor contains one light emitter with 775, 810, 850 and 910 nm wavelengths and one detector with 3 segments (SI-photodiodes). The pre-calibrated emitter and detector optodes were fixed in a probe holder to ensure the inter-optode distance of 50 mm. The chosen path-length of the NIRO was 19 cm, and the optical path-length factor was 3.8 [26]. All measured data were stored electronically at a sample time of 2 seconds (0.5 Hz) for subsequent analysis, while demographic data were noted. The measurements were performed by trained study personnel who were not involved in the care of the newborns in the delivery room.

After 12–24 hours of life, a second measurement was performed over 15 minutes on the maternity ward or in the neonatal intensive care unit (NICU).

### Ethics

The study design was approved by the hospital’s Ethic Committee (Centre of Ethics, University of Zurich). Due to the emergency situation leading to perform vacuum delivery due to fetal distress, oral consent was obtained by the present father for the first measurement and a formal written parental consent was obtained thereafter for the second measurement. All parents asked for permission agreed.

### Statistical analysis

Mean values for THI, TOI, pre-ductal tcSaO_2_ and HR were calculated for the first 0–5, 5–10 and 10–15 minutes, and for the measurement at 12–24 hours of life. These mean values were compared by Wilcoxon Mann Whitney test with those of 19 healthy newborns delivered by elective cesarean section [13]. The analysis was performed by using IBM SPSS, Version 18, Inc, Chicago, IL.and Matlab, the mathworks, version 7.7, Natick, MA.

A sample size of 15 in the vacuum group was calculated based on the hypothesis that a control group included 19 infants born after elective cesarean section without fetal distress [13] and there was an effect size of 1, i.e. the difference in TOI between the two groups was the same or larger than the standard deviation within the groups (Power 80%, alpha 5%).

## Results

The clinical data of the 15 newborn infants delivered by vacuum extraction and of the 19 infants born by uncomplicated elective cesarean section are shown in Table 1. There was no statistically significant difference between the groups regarding gestational age, birth weight, and Apgar scores. Infants after vacuum extraction had a significantly lower pH in the umbilical arterial blood and showed a tendency for low base excess values and high lactate levels. In the vacuum group, 4 infants required supplemental oxygen due to persisting cyanosis with a FiO_2_ of 0.25 during the first 10 minutes, and one infant needed intermittent bag and mask ventilation for the first 2 minutes of life. No infant in the cesarean section group required supplemental oxygen or bag and mask ventilation. Four infants were admitted to NICU after vacuum delivery due to respiratory distress (RDS), hypothermia, hypoglycemia or feeding difficulties and none after cesarean section.

**Table 1:** Clinical characteristics of the study group

	**Vacuum (n = 15)**		**C-section (n = 19)**	
	**Median**	**Range**	**Median**	**Range**
Gestational age (wk)	40	38-41	38	37-40
Birth weight (g)	3390	2480-4200	3130	2520-4190
APGAR 1 minute	8	1–9	8	7–9
5 minutes	9	6-10	9	8-10
Umbilical artery pH	7.23	7.12 - 7.31	7.28	7.2-7.38
BE	−6.53	-11.3-(−3.0)	−0.2	-2.2-2.4
Lactat (mmol/l)	5.33	3.4-7.8		
Hospitalization on NICU (n)	4		1	

**Figure 1:**
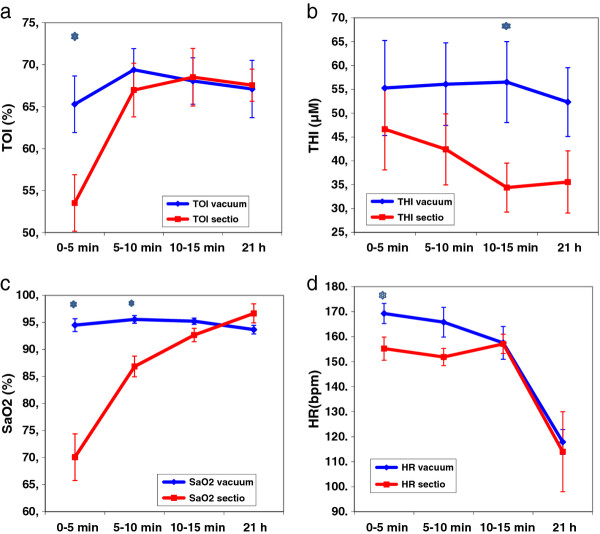
**a) TOI b) THI c) SaO**_**2 **_and d) HR, during the first 15 minutes (min) and 12–24 hours (mean 21 hrs) after birth (mean values ± SEM) for the vacuum group (blue) vs sectio (red); * markes significant differences.

The median age at start of NIRS measurements was 2 minutes after birth (range 0 to 4 minutes), and SpO_2_ measurements were reliable within 1 minute after the sensor was placed. The values of TOI, THI, SaO_2_ and HR for the first 0–5, 5–10 and 10–15 minutes and for the measurement at 12–24 hours (median 21 hours) of life are given in Table 2 and Figure 1. Four out of 15 measurements in the vacuum group were omitted due to movement artifacts. The mean values of TOI in the vacuum group (*vs.* cesarean section) rose from 65% at 0–5 minute, to 69% at 5–10 minute, and reached a steady state at 15 minutes (Figure 1a). TOI was significantly lower (54%) at 0 to 5 min and lower at 5–10 min (66%) in the section group and reached the same level as the vacuum group at 10 to 15 min and at 21 hours. THI was around 55 μM during the whole measuring period and significantly higher at 10 to 15 minutes than in the section group (Figure 1b). The SaO_2_ level in the first 10 min and the HR in the first 5 minutes were significantly higher in the vacuum group (Figure 1c and d).

**Table 2:** **Mean values ± SEM of HR, SaO2, TOI and THI during the first 15 minutes (min) and 12–24 hours (mean 21 h) after birth, mean difference, 95% confidence interval (CI) of the difference p-value (significant p < .05 by Mann–Whitney *U *** **test)**

		**0-5 min**			**5-10 min**			**10-15 min**			**21 h**		
		**Mean ± SEM**	**95% CI**	**P-value**	**Mean ± SEM**	**95% CI**	**P-value**	**Mean ± SEM**	**95% CI**	**P-value**	**Mean ± SEM**	**95% CI**	**P-value**
	Vacuum	65 ± 3			69 ± 8			68 ± 3			67 ± 6		
**TOI (%)**	C-section	53 ± 3			67 ± 3			69 ± 4			68 ± 2		
	Difference	11.6 ± 6.3	−1.4 to 24.9	**0.03**	2.4 ± 4.6	−7.1 to 11.9	0.35	−0.5 ± 5	–10.7 to 9.8	0.99	−0.5 ±4.4	−9.8 to 9	0.67
	Vacuum	55 ± 10			56 ± 9			56 ± 9			52 ± 7		
**THI (μM)**	C-section	47 ± 9			42 ± 7			34 ± 5			36 ± 7		
	Difference	8.6 ± 16	–25.1 to 42.4	0.27	13.7 ± 11.8	−10.5 to 37.8	0.17	22.1 ± 9.3	−2.9 to 41.2	**0.04**	16.8 ± 10.2	−5.1 to 38.6	0.11
	Vacuum	169 ± 4			166 ± 18			158 ± 7			118 ± 5		
**HR (/min)**	C-section	155 ± 5			152 ± 3			157 ± 4			114 ± 16		
	Difference	14.1 ± 9.1	−5.2 to 33.3	0.19	13.9 ± 6.4	−0.7 to 27.1	0.06	−0.4 ± 7.1	−14.2 to 15	0.83	3.9 ± 12.4	−24.3 to 32.0	0.69
	Vacuum	95 ± 1			95 ± 2			95 ± 1			94 ± 1		
**SaO**_**2**_**(%)**	C-section	70 ± 4			87 ± 2			93 ± 1			97 ± 2		
	Difference	24.4 ± 828	6.9 to 41.8	**0.001**	9.7 ± 2.6	3.3 to 14.1	**0.002**	2.6 ± 1.7	−0.9 to 6.1	0.18	−3.0 ± 1.7	−6.8 to 0.8	0.19

## Discussion

This study shows a higher THI in infants delivered by vacuum after fetal distress than control infants delivered by cesarean section without fetal distress, but this difference was only significant after 10–15 minutes after birth. We also found a higher TOI and SaO2 for the first 10 minutes in the vacuum group compared to the control group.

This finding is in contrast to previously published studies that did not show a difference in TOI nor THI between infants after elective cesarean section and vaginal delivery [11,12,15].

Few studies have investigated changes in cerebral oxygenation and hemodynamics in neonates immediately after birth, and during the first hours of life [6,7,9-17,27]. NIRS studies in the immediate postpartum period showed a rapid increase in cerebral oxygenation after birth with a decrease after few hours, but the influence of the mode of delivery on these changes has been discussed controversially [9-12,15].

Dani et al. performed a study looking at changes between two to five hours after birth. They found that, independently of the delivery mode, O_2_Hb and CBV decreased approximately at four and five hours of life. They found a decrease in CBV of about 10% at the fifth hour of life, and they attributed this decrease to a reduction of cardiac output after delivery, and also to the increase of the left-to-right-shunt through the arterial duct, which progressively increases in the first hours of life with decreasing pulmonary resistance. The values of mixed cerebral oxygenation ranged from 64% to 72% in infants born by vaginal delivery, and from 64% to 70% in infants born by cesarean section [12]. Urlesberger et al. published two studies about cerebral oxygen saturation during birth transition in term infants [14,15], one comparing vaginal with elective cesarean delivery [15]. They reported TOI (oxygenated/total haemoglobin) increasing from 50% at 3 minutes after birth to 80% at 8 minutes not depending on the mode of delivery. These values are considerably higher than ours which is most likely due to different instrumentation using different hardware and different algorithms.

The higher THI and TOI after vacuum extraction in our study can be explained by several factors. The indication for vacuum extraction was fetal distress and therefore these infants are a selection of highly stressed infants with high catecholamine levels which lead to accelerated heart rate, increased cerebral perfusion and increased tissue oxygenation. Other factors may be local forces by the vacuum, pain and instrumental design.

It has been shown that in vaginally delivered neonates the catecholamine surge was significantly different compared to neonates delivered by cesarean section, with higher serum epinephrine and norepinephrine concentrations in the vaginally delivered group [28]. These concentrations were found to be very high at the moment of birth, and to fall during next 2–4 hours after an uncomplicated vaginal delivery [27,29-32]. Isobe et al. speculated that in a vaginal delivery, many catecholamines are released and act to constrict the peripheral vessels, and to initially increase CBF. Later on, the arterioles remain dilated for a relatively long time due to this effect, which may be the reason for continued high levels of TOI after vaginal delivery [11]. However, norepinephrine is able both to constrict (via alpha-receptors) and to dilate (via beta-receptors) cerebral vessels [33]. This could also explain the higher THI values we observed in the group of infants delivered by vacuum extraction. It is known, that NIRS is particularly sensitive to small blood vessels and that the TOI represents the oxygen saturation of all the haemoglobin in these vessels. Approximately 70-75% of this haemoglobin is in the venous, approximately 20-25% in the arterial and the rest in the capillary compartment [34-36]. If only the arterioles remain dilated, this would consequently account for only a small increase in THI. But a dilation of the arterioles also means that the resistance of the vessels is reduced. Consequently the blood flow is increased, which explains the high TOI values (washout effect) and since this also leads to a higher pressure within capillaries and veins, i.e. all the vessels will open up, not only the arterioles, this leads to a significantly increased THI.

The mode of delivery has also a transitory effect on cerebral vascular resistance [8,32]. A study in which peripheral blood vessel resistance was measured reported that peripheral constriction remained higher for up to 2 hours after birth in infants delivered vaginally when compared to a cesarean section group [32]. The cerebral vascular resistance showed higher values 1 hour after birth in newborns after cesarean section, but after 24 hours of life the values were equal for the vaginally delivered group [30]. In a recent study in healthy term neonates Noori et al. speculated, that the reduction in cerebral blood flow (CBF) 8 minutes after normal vaginal delivery is likely due to an increase in arterial O_2_ content, Patent ductus arteriosus (PDA) shunting or both [16]. The authors concluded that the offset of the left-to-right PDA shunt and the full compensatory increase in left-ventricular output might not yet be in place soon after birth in this group of healthy term newborns after vaginal delivery [16]. Assuming this as a physiologic response with a consecutive drop or plateau in cerebral blood flow after approximately 8 minutes after birth found by several groups [11-16], we can only speculate whether an earlier onset and longer persistent increased left ventricular output in infants after vacuum delivery is responsible for the remaining high THI values. The other explanation for higher THI values could also be fetal distress during labor. Towner et al. suggested, that a substantial proportion of the morbidity associated with operative vaginal delivery may be due to an underlying abnormality of labor [18]. Nevertheless, although there seems to be no differences in the long-term cognitive development , the clinician must be aware of short-term morbidities such as pain, feeding difficulties or jaundice after vacuum extraction, which can occur within the first 10 hours of life [37,38] or more serious morbidities such as subgaleal hematomas or intracranial haemorrhage [18,19].

Although the infants of the vacuum group were born after fetal distress, this distress had only a short effect on cerebral hemodynamics lasting less than 24 hours when compared to healthy infants born after elective cesarean section. There was still a trend for higher THI after 24 hours, but this oberservation was not statistically significant. This finding is important as from the clinical point of view many of these studied infants born after vacuum delivery showed a prolonged postnatal adaptation, and 4 infants had to be admitted to NICU. Furthermore, infants in our vacuum group showed a lower cord blood pH, a tendency for low base excess values and high lactate levels, a phenomenon, which was also shown in a study by Salamalekis et al. [37].

We found higher SaO_2_ levels in the vacuum group during the first 5 minutes compared to the control group born by cesarean section, which can also explain higher TOI and THI in the first 10 minutes. This difference in SaO_2_ immediately after birth is in agreement with the published [2,4,5]. Harris et al. speculated that the difference was due to the increased amount of lung fluid after caesarean section [2]. 4 infants in our study group required intermittend oxygen during the first 10 minutes. The initial decision on whether to initiate oxygen supplementation was based on clinical evaluation only due to persisting cyanosis. But interestingly there was no difference between these infants with or without oxygen in reaching SaO_2_ levels > 90% in the first 5 minutes. This finding is in accordance with previous studies in asphyxiated newborns with no difference in time to reach SaO_2_ levels > 90% between the groups receiving oxygen or room air for resuscitation [39,40]. A limitation of the present study is that our study group is very small with 11 infants and we therefore could miss some more differences in our parameters. We did not measure the haematocrit values in our newborns, which may influence cerebral oxygenation. Another question could be if the differences between our groups are due to vacuum extraction by itself or due to fetal distress. Vacuum extraction is often associated with caput succedaneum (edema of the scalp) or with a cephalhematoma [18-21]. In both situations there is a potential for significant volume trapping which is not available for cardiac output, and therefore also not for cerebral perfusion. The physicians taking care of these babies in the delivery room may balance reasons about whether a particular newborn with or without clinical signs of hypovolemia needs a volume therapy or not. NIRS may be useful for guiding volume therapy in these infants at risk.

Since the light of NIRS penetrates the skin and skull before it reaches the brain, one potential issue may be that the TOI and THI are influenced by these superficial tissues. In particular this may be a problem in case of a haematoma below the NIRS sensor. Therefore, we always placed the NIRS sensor on the right forehead, where there never was a haematoma. The NIRO 300 that we have employed as NIRS instrument has two modes of operation: 1) the approach based on the modified Lambert Beer law, which provides relative changes in the concentration of O_2_Hb and HHb, which are known to be affected by superficial tissue. For this reason, these data have not been presented here. 2) The other approach is based on spatially resolved spectroscopy [25], which measures the decrease in intensity with the source detector distance and which was demonstrated by Franceschini [41] to remove the influence of superficial tissue. The TOI and THI values are calculated according to spatially resolved spectroscopy and consequently reflect oxygenation and blood volume of the brain unaffected by superficial tissue. TOI is the NIRO 300’s most reliable parameter, because it is a ratio and hence the absolute value of the scattering coefficient, which cannot be measured by the NIRO 300, and other factors reducing the reliability cancel out. By assuming a reasonable value of the scattering coefficient, the total haemoglobin concentration (THI) can be determined as an absolute value. Since this parameter requires an assumption, it is less reliable than the TOI. On the basis of measurements in single subjects, an error in the assumption will propagate directly to the THI value. Unless there are factors affecting the scattering coefficient systematically and differently for the two groups, for a group of subjects in average as for any statistical mean, the error should average out or at least be substantially reduced. Consequently when a difference in THI is found between two groups, this is probably a fairly reliable result.”

## Conclusion

In summary, our study shows that the cerebral oxygenation and perfusion in newborns delivered by vacuum extraction following fetal distress is disturbed in the first 15 minutes compared to newborns delivered by elective cesarean section.

## Abbreviations

CBF: Cerebral blood flow; CBV: Cerebral blood volume; O2Hb: Oxygenated hemoglobin; HHb: Deoxygenated hemoglobin; Hb: Total hemoglobin; HR: Heart rate; NIRS: Near-infrared spectroscopy; NICU: Neonatal intensive care unit; PDA: Persistent ductus arteriosus; RDS: Respiratory distress syndrome; SaO2: Arterial oxygen saturation; TOI: Tissue oxygenation index = 100* O_2_Hb/(O_2_Hb + HHb); THI: Tissue hemoglobin index = O_2_Hb + HHb.

## Competing interests

The authors declare that they have no competing interests.

## Authors’ contributions

All of the authors contributed to the study planning, discussion and interpretation of data. All the authors read and approved the final manuscript. TK was the principle investigator for this work. She wrote the first draft of the manuscript. The data collection and data management were performed by TK and RN. MW performed the statistical analysis.

## Pre-publication history

The pre-publication history for this paper can be accessed here:

http://www.biomedcentral.com/1471-2431/14/21/prepub
